# Socioeconomic factors predicting outcome in surgically treated carpal tunnel syndrome: a national registry-based study

**DOI:** 10.1038/s41598-021-82012-x

**Published:** 2021-01-28

**Authors:** Malin Zimmerman, Evelina Hall, Katarina Steen Carlsson, Erika Nyman, Lars B. Dahlin

**Affiliations:** 1 Department of Translational Medicine – Hand Surgery, Lund University, Skåne University Hospital, Jan Waldenströms gata 5, 205 02 Malmö, Sweden; 2 Department of Hand Surgery, Skåne University Hospital, Jan Waldenströms gata 5, 205 02 Malmö, Sweden; 3 Department of Clinical Sciences, Malmö, Lund University, Lund, Sweden; 4 Department of Biomedical and Clinical Sciences, Linköping University, Linköping, Sweden; 5 Department of Hand Surgery, Plastic Surgery and Burns, Linköping University Hospital, Linköping, Sweden

**Keywords:** Peripheral nervous system, Peripheral neuropathies

## Abstract

The study aimed to investigate socioeconomic factors in patients with carpal tunnel syndrome (CTS) and to evaluate their impact on outcome following open carpal tunnel release (OCTR). Data from the National Quality Register for Hand Surgery were combined with socioeconomic data (marital status, education level, earnings, migrant status, occupation, sick leave, unemployment, and social assistance) from Statistics Sweden to evaluate OCTRs performed from 2010 to 2016 (total 10,746 OCTRs). Patients completed QuickDASH (short version of Disabilities of Arm, Shoulder and Hand) questionnaires preoperatively (n = 3597) and at three (n = 2824) and 12 months (n = 2037) postoperatively. The effect of socioeconomic factors on QuickDASH scores was analyzed with linear regression analysis. Socioeconomically deprived patients scored higher on the QuickDASH on all occasions than patients with higher socioeconomic status. Being widowed, having a low education level, low earnings, immigrant status, frequent sick leave and dependence on social assistance all increased the postoperative QuickDASH score at 12 months. The change in total score for QuickDASH between preoperative and 12 months postoperatively did not vary between the groups. We conclude that such factors as being widowed, having a lower education level, low earnings, immigrant status, frequent sick leave and social assistance dependence are associated with more symptoms both before and after OCTR for CTS, but these factors do not affect the relative improvement in QuickDASH.

## Introduction

Carpal tunnel syndrome (CTS) is the most common peripheral nerve compression lesion, affecting 3% of the general population^1^ with known co-morbidity, such as diabetes, hypothyroidism and neuropathy. The risk factors for the development of CTS and poor treatment outcome include smoking, exposure to vibration, repetitive work and force as well as combined exposures^2^. The standard treatment for permanent symptoms or disability is surgery with open carpal tunnel release (OCTR). This is a common, and usually successful, procedure but incurs high costs for society due to the large number of cases^3^.

Socioeconomic status (SES) is defined as the social standing of an individual or group in relation to others, often measured by indicators, such as education, income and/or occupation^4^. SES is a major determinant of morbidity and mortality in the general population^5^. The evidence regarding SES in the development of CTS stays undetermined, as few studies, with conflicting results, have been conducted. A higher level of education and a higher annual income have previously correlated positively to both self-reported and diagnosed CTS^2^, possibly due to more awareness of the condition amongst these individuals. In contrast, another study shows that CTS is more common in socioeconomically deprived groups^6^, possibly as result of occupational factors.

Less is known regarding the impact of socioeconomic factors on general recovery and outcome after OCTR. A systematic review found poor preoperative functional status, family support and mental health, and less supportive work organization, exposure to bending of the hands, heavy lifting and highly repetitive work to be risk factors for a prolonged period before return to work after OCTR^7^.

We hypothesized that socioeconomically deprived groups would often present with CTS and have worse outcomes following OCTR, but that the difference between pre- and post-operative measures may not be equally strongly correlated with socioeconomic factors. Hence, we aimed to investigate the association between socioeconomic factors, presence of bilateral CTS and outcome after OCTR for primary CTS, as measured by QuickDASH.

## Methods

A retrospective observational study was performed with data on CTS cases (ICD-10 diagnosis code G560) treated with OCTR (KKÅ97 operation code ACC51) collected from the Swedish National Quality Registry of Hand Surgery (HAKIR; hakir.se)^8^ between February 2010 and December 2016. All seven university hand surgery clinics in Sweden and two private hand surgery clinics report to the registry. People with a Swedish Personal Identity Number, who are able and willing to provide informed consent, are included in the registry. If the patient is re-operated on within one year of the primary surgery, no Patient Related Outcome Measurement (PROM) is sent to the patient. Patients under the age of 18 years and patients with incorrect data from HAKIR were excluded. Patients with bilateral CTS operated on within the study period were included as two individual cases.

The Swedish version of the QuickDASH (shortened version of the DASH; Disabilities of the Arm, Shoulder and Hand)^9^ was used to evaluate disability preoperatively and at three and 12 months postoperatively. QuickDASH comprises eleven items, and gives a total score ranging from 0 to 100, where a higher score represents greater disability. The questionnaires were sent by e-mail or regular mail with one reminder.

Data from HAKIR, together with data on diabetes status from the Swedish National Diabetes Registry (NDR; ndr.nu), were combined with socioeconomic data from Statistics Sweden (SCB; scb.se)^10^. From SCB, the following variables were retrieved: marital status, education level, earnings, migrant status, occupation, sick leave, days of unemployment, and social assistance. Marital status, type of work; manual/non-manual, and level of education were recorded for the year of surgery. Marital status included single, married/registered partner, divorced and widowed. Data concerning outcome in patients with and without diabetes have been presented previously^10^.

Data regarding earnings were available from 1990 to 2016. Earnings were indexed to December 2016 using the consumer price index (available from Statistics Sweden, www.scb.se). For reference, a cost-indexed basic amount in 2016 was SEK 44,300 and 1 euro was worth approximately SEK 9·47. To measure long-term earnings, we calculated the mean earned income per year, including the years when the patient was between 30 and 65 years of age; i.e., working years, retirement age is commonly 65 years in Sweden. From these mean annual earnings, a binned variable was created based on percentiles.

Sick leave was calculated as net days; one day with 100% sick leave counts as one net day, one day with 50% sick leave counts as a 0·5 net day etc.. In Sweden, since 1991, the social security system has been organized in such a way that the employer pays for the first 14 days sick leave. Thus, data were only available for the days after the first 14 days. One is entitled to paid sick leave after being employed for a minimum of six months. A binned variable was created based on how many days the patient had been on sick leave (mean per year above 20 years of age). Data were available from 1994 to 2016.

Data on unemployment were available from 1992 to 2016. Mean number of days of unemployment per year was calculated. Data on paid social assistance (individualized) were available from 1990 to 2016. A binned variable was created: those who had never received social assistance; those who had received it once; and those who had received it more than once.

Education level was divided into three groups. These groups correspond to the International Standard Classification of Education (ISCED)^11^ as follows: primary—ISCED 0, 1 and 2 (≤ 9 years of education, compulsory school); upper secondary—ISCED 3 (10–12 years of education); and tertiary—ISCED 4, 5 and 6 (> 12 years of education).

### Statistics

Continuous data are presented as median [interquartile range IQR]. Categorical variables were created from continuous variables, based on their distribution, to investigate non-linear correlations and for convenience of interpretation. The Mann–Whitney U-test was used to calculate comparisons between two groups and the Kruskal–Wallis Test for more than two groups. In subsequent pairwise comparisons, *p* values were adjusted using the Bonferroni correction for multiple tests. Nominal data are presented as numbers (%) and compared using the Chi-squared test.

A multivariate linear regression analysis was performed to investigate the association between socioeconomic factors and QuickDASH scores. In model 1, each variable was separately analysed. In model 2, all variables were adjusted for sex, diabetes, and age at surgery. Model 3a included all variables. The reduced model 3b^12^ included all variables with a *p* value < 0·3 in model 3a. In the reduced model 3c, all variables with a *p* value < 0·1 in reduced model 3b were included.

Each treated hand was considered a separate statistical entity. A *p* value of < 0·05 was judged statistically significant. All calculations were performed using IBM SPSS Statistics version 24 or 25 (SPSS Inc., Chicago, IL).

### Ethics

This study was approved by the Regional Ethical Review Board in Lund, Sweden (2016/931, 2018/57 and 2018/72). Appropriate permissions were obtained from HAKIR and SCB to use the data from the registries.

### Role of the funding source

The study sponsors were not involved in study design, in the collection, analysis and interpretation of data, in writing the report or in the decision to submit the paper for publication. MZ (corresponding author) had full access to the data in the study and final responsibility for the decision to submit the study for publication.

## Results

During the study period, 10,770 OCTRs were registered in HAKIR. Of these, 22 were under the age of 18 and were excluded; a further two cases were excluded due to incorrect data from HAKIR. The study population comprised 9029 people, of whom 1717 (19%) had bilateral OCTR resulting in 10,746 operated cases. Preoperative QuickDASH scores among all cases were median 52 [IQR 34–66], at three months postoperative 23 [9–43] and at 12 months postoperative 16 [5–39] (Table 1). Preoperative population characteristics are presented in Table 1.

**Table 1:** Cases treated with open carpal tunnel release divided into age categories.

	18–35 years (n = 1242)	36–45 years (n = 1635)	46–55 years (n = 2452)	56–65 years (n = 2280)	66–75 years (n = 1617)	> 76 years (n = 1520)	*p* Values	All cases (n = 10,746)
Sex, female n (%)	964 (78)	1179 (72)^a^	1708 (70)^ns^	1443 (63)^f^	891 (55)^h^	965 (64)^j^	**< 0·0001**	7150 (67)
Born outside Sweden n (%)	183 (15)	305 (19)^a^	512 (21)^ns^	409 (18)^e^	192 (12)^h^	166 (11)^ns^	**< 0·0001**	1767 (16)
Bilateral OCTR n (%)	214 (17)	317 (19)^a^	387 (16)^d^	355 (16)^ns^	219 (14)^h^	226 (15)^ns^	**< 0·0001**	1717 (16)
Employed all years* n (%)	582 (47)	468 (29)^b^	737 (30)^ns^	772 (34)^e^	65 (4)^h^	0 (0)^j^	**< 0·0001**	2624 (24)
Never employed n (%)	74 (6)	68 (4)^a^	79 (3)^ns^	79 (4)^ns^	71 (4)^ns^	313 (21)^j^	**< 0·0001**	684 (6)
Highest education level n (%)	942 (76)	945 (58)^b^	976 (40)^d^	902 (40)^ns^	632 (39)^ns^	386 (25)^j^	**< 0·0001**	4783 (45)
Sick days/employed year median [IQR]	9 [3–36]	12 [4–35]^ns^	12 [3–42]^ns^	11 [2–37]^e^	7 [0–39]^h^	0 [0–7]^j^	**< 0·0001**	9 [1–35]
Days as unemployed/year median [IQR]	N/A	19 [4–40]	8 [0–29]^d^	0 [0–13]^f^	0 [0–3]^h^	0 [0–0]^j^	**< 0·0001**	0 [0–18]
Ever received social assistance n (%)	484 (39)	823 (50)^b^	1032 (42)^d^	746 (33)^f^	295 (18)^h^	104 (7)^j^	**< 0·0001**	3484 (32)
Mean annual earnings, 1000 SEK median [IQR]	184 [0–287]	240 [158–310]^b^	217 [132–278]^d^	228 [142–296]^ns^	196 [110–281]	115 [19–197]^j^	**< 0·0001**	206 [98–281]
Preoperative QuickDASH median [IQR]	52 [39–68]	50 [32–66]^a^	50 [34–66]^ns^	50 [32–66]^ns^	48 [32–64]^ns^	57 [41–70]^j^	** < 0·0001**	52 [34–66]
Postoperative QuickDASH at 3 months median [IQR]	18 [7–34]	20 [11–36]^ns^	23 [11–43]^ns^	20 [9–39]^e^	20 [9–39]^ns^	30 [14–52]^j^	**< 0·0001**	23 [9–43]
Postoperative QuickDASH at 12 months median [IQR]	14 [5–34]	11 [5–34]^ns^	16 [5–36]^ns^	14 [2–36]^ns^	16 [5–36]^ns^	30 [9–50]^j^	**< 0·0001**	16 [5–39]
Change in QuickDASH score 0–12 months median [IQR]	27 [14–45]	25 [11–38]^ns^	27 [12–41]^ns^	27 [14–40]^ns^	23 [11–39]^ns^	20 [5–41]^ns^	0·085	25 [11–41]

*between 1990 and 2016. Only years > 20 years of age. Values are median and [IQR] or numbers (%). OCTR = open carpal tunnel release.

Days as unemployed calculated as days as unemployed/years above 20 years of age. There were too few valid cases (n = 13) in the group 18–35 years to calculate days as unemployed.

^a^*p* < 0·05 between group 1 and 2, ^b^*p* < 0·0001 between group 1 and 2, ^c^*p* < 0·05 between group 2 and 3, ^d^*p* < 0·0001 between group 2 and 3, ^e^*p* < 0·05 between group 3 and 4, ^f^*p* < 0·0001 between group 3 and 4, ^g^*p* < 0·05 between group 4 and 5, ^h^*p* < 0·0001 between group 4 and 5, ^i^*p* < 0·05 between group 5 and 6, ^j^*p* < 0·0001 between group 5 and 6. ns = non-significant. Kruskal Wallis test with subsequent Bonferroni corrections were used to calculate statistical significance.

### Responders vs. non-responders

Preoperatively, 3597/10,746 cases (33%) responded to the PROM. At three months postoperatively the response rate was 2824/10,010 (28%) and at 12 months 2037/8297 (25%). Sex distribution did not differ between responders and non-responders on any occasion. There were no significant age differences between responders and non-responders preoperatively. Responders were older than non-responders at both 3 (median 59 [IQR 49–71] years vs. 54 [43–67] years; *p* < 0·0001) and 12 months after surgery (60 [49–72] vs. 55 [44–67]; *p* < 0·0001).

### Age

Younger patients had higher education levels and more often received social assistance (Table 1). QuickDASH scores were highest among the oldest patients on all three occasions (Table 1).

### Marital status

**Figure 1:**
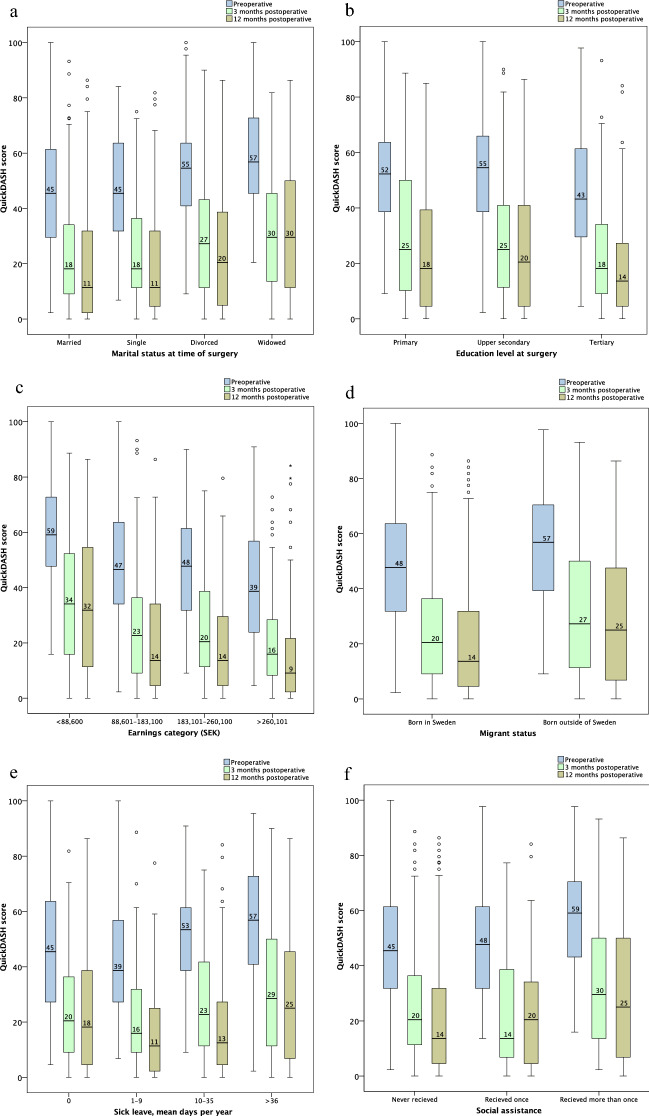
QuickDASH scores. (**a**) Groups based on marital status. (**b**) Groups based on education level. (**c**) Groups based on earning category (mean earnings per year between 20 and 70 years of age). (**d**) Groups based on migrant status. (**e**) Groups based on mean sick leave days per year. (**f**) Groups based on social assistance.

Data were missing in 40/10,746 (0·4%) cases. QuickDASH scores were highest on all occasions among the divorced and widowed (Fig. 1, Supplementary Table S1 online). In the linear regression analysis, being widowed increased the 12-month postoperative QuickDASH score by 8 points (supplementary Table S7 online).

### Education level

Data were missing in 186/10,746 (1·7%) cases. Earnings were highest in the group with the highest education level, and the proportion of patients who had ever received social assistance was lowest in this group (Supplementary Table S2 online). The patients with the highest education level scored lowest in the QuickDASH on all occasions, but there were no differences in change in QuickDASH score between groups (Fig. 1, Supplementary Table S2 online). In the linear regression analysis, the highest education category predicted a QuickDASH score 7 points lower at 12 months (Supplementary Table S7 online).

### Earnings

There were more men in the highest earning group than in the other groups. The highest earning group also had the highest proportion of tertiary education and the smallest proportion of cases born outside Sweden (Supplementary Table S3 online). The highest earning group had the lowest QuickDASH scores on all occasions and the lowest earning group had the highest QuickDASH scores on all occasions, but the change in total QuickDASH score did not differ between the groups (Fig. 1, Supplementary Table S3 online).

In the linear regression analysis, being in the high earning group reduced the QuickDASH score at 12 months by 12 points (Supplementary Table S7 online).

### Migrant status

There were more women in the group of cases born outside Sweden than in the group born in Sweden (Supplementary Table S4 online). Among cases who were not born in Sweden, 1023/1767 (58%) had received social assistance at least once compared to 2461/8979 (27%) cases born in Sweden (*p* < 0·0001; Supplementary Table S4 online). Immigrants scored higher in the QuickDASH on all occasions (Fig. 1d; Supplementary Table S4 online). In the linear regression, being an immigrant increased the postoperative QuickDASH score at 12 months by 6 points (supplementary Table S7 online).

### Manual occupation

Data were missing in 5147/10,746 (48%) cases. We found no statistically significant effects of manual occupation on postoperative QuickDASH score at 12 months (Supplementary Table S7 online).

### Sick leave

The group with the highest number of sick days contained more women and more cases born outside Sweden than the other groups. The group with the highest number of sick days scored highest in QuickDASH on all occasions (Fig. 1e, Supplementary Table S5 online). The mean length of sick leave the year before surgery was 19 days, and 25 days the year after surgery. The year before surgery, 435 cases (4·0%) had > 180 days of sick leave. The same year as surgery, 713 (6·6%) cases had > 180 days of sick leave. In the linear regression analysis, being in the group with the highest number of sick days was associated with a postoperative QuickDASH score at 12 months that was 5 points higher (Supplementary Table S7 online).

### Days unemployed

Data were missing in 2432/8314 (23%) cases. No significant results were found for unemployment in the linear regression analysis (Supplementary Table S7 online).

### Social assistance

Women received more social assistance than men. Cases who had ever received social assistance scored higher than cases who had never received it, on all occasions (Fig. 1f, Supplementary Table S6 online). In the linear regression analysis, having received social assistance more than once increased the postoperative QuickDASH score at 12 months by 10 points compared to never having received any social assistance (Supplementary Table S7 online).

### Reduced regression models

Factors with a *p* value of < 0·3 in the original model were included in the first reduced model, with the 12-month postoperative QuickDASH score as the dependent variable (Supplementary Table S8 online). The model was reduced in one more step by including only the factors with a *p* value of < 0·1 in the first reduced model, producing the final reduced model (Table 2).

**Table 2:** Reduced model 3c.

	Coefficient (95% CI)	*p* Value
*Marital status*		
Not married/married/divorced (reference)		
Widowed	6·0 (2·7–9·2)	< 0·0001
*Level of education*		
Low/middle (reference)		
High	− 4·0 (− 6·0 to − 2·0)	< 0·0001
*Earnings (mean/year)*		
≤ 98,100 (reference)		
98,101–202,600 SEK	− 8·1 (− 10·8 to − 5·3)	< 0·0001
202,601–281,000 SEK	− 10·6 (− 13·5 to − 7·7)	< 0·0001
> 281,000 SEK	− 12·2 (− 15·2 to − 9·1)	< 0·0001
*Migrant status*		
Born in Sweden (reference)		
Born outside of Sweden	7·4 (4·5–10·2)	< 0·0001
*Sick leave*		
0 days/10–35 days (reference)		
1–9 days	− 6·2 (− 8·5 to − 4·0)	< 0·0001
> 36 days	5·1 (2·4–7·7)	< 0·0001
*Social assistance*		
Never received/received once (reference)		
Received more than once	8·0 (5·5–10·5)	< 0·0001

Multivariate linear regression analysis of QuickDASH at 12 months. All variables with *p* value < 0.1 in reduced model 3b are included.

Excluded variables: age at surgery, sex, diabetes at surgery, manual/non-manual occupation, and unemployment. Coefficients are unstandardized. CI; confidence interval.

In the final reduced model, widowed patients scored higher on the QuickDASH 12 months postoperatively than patients who were not widowed. Tertiary education and higher earnings predicted lower 12-month postoperative QuickDASH scores. Patients who were born outside Sweden scored higher in the QuickDASH at 12 months postoperatively, as did patients who had received social assistance more than once. Sick leave for 1–9 days predicted a lower 12-month postoperative QuickDASH score, whereas the highest category (> 36 days), predicted a higher QuickDASH score at 12 months postoperatively.

## Discussion

The present findings suggest that there is an association between socioeconomic factors, experienced disability, and hand function before and after OCTR due to primary CTS. These associations remained after adjusting for other common prognostic factors, such as age, sex and diabetes^13–15^. Consistent with the original hypothesis, in which a higher level of education and higher earnings positively correlate to CTS^2^, our results demonstrate that lower socioeconomic status is associated with worse rated disability both pre- and post-operatively. However, we found no differences in improvement through surgery, as measured by the change in QuickDASH. This implies that patients with different socioeconomic backgrounds recover and benefit equally from surgery, though consistently rating their symptoms and functional impairment differently.

Clement et al. recently investigated threshold values in the QuickDASH in 937 OCTRs. They found that a change of ≥ 20 points and a postoperative QuickDASH score at 12 months of ≤ 34 points correspond well with patient satisfaction^16^. Applying these thresholds to our population shows that most patients were satisfied with surgery, regardless of socioeconomic status. However, the question of why some factors are associated with more perceived disability remains. When analysing QuickDASH, it seems important to not only look at the postoperative scores, but also to analyse how the total score has changed from preoperative to postoperative.

It is possible that, in our population, groups with lower socioeconomic status perceived their general health to be worse than groups with higher socioeconomic status. Even though the surgical procedure had the same effect in all groups, patients with lower socioeconomic status still perceived their disability after surgery as worse. Although not used in this study, the 12-item Short Form (SF-12)^17^ is commonly used to assess general health, and DASH scores correlate with SF-12 scores, particularly in the physical health domains^18^. Furthermore, the individual patient´s “sense of coherence”, i.e. a theory that uses a salutogenic approach to health^19^, may also be relevant, as has been observed in major hand injuries^20^. It remains to be evaluated whether sense of coherence, as estimated in patients with CTS and OCTR^21^, is associated with socioeconomic factors and outcome of surgery. However, bearing in mind the present results, the individual’s sense of coherence may be an important factor for the perceived disability before and after surgery for CTS, while the relative improvement in QuickDASH is not affected.

Education is an important influencer of health outcomes, through knowledge, literacy, and problem-solving abilities etc ^4^. It is possible that individuals with a higher level of education have a greater awareness of CTS and seek medical care more often and earlier in the course of the disease. This might affect treatment results, as long-standing CTS may result in irreversible nerve damage.

The group with the lowest earnings scored their symptoms highest. Retired patients are included in this group. We adjusted for this fact by calculating mean earned income for the years when the patient was aged between 30 and 65 years. However, this result persisted in the linear regression analysis when adjusting for age. It cannot be completely explained by sex differences (there were more men in the high-earning group), since the results persisted when adjusting for sex. One might suspect that occupational factors, such as more manual occupations in the low-earning group, could influence the results. However, our data did not support the hypothesis that occupational factors are a significant factor for disability before or after surgery.

One striking finding is that 32% of the patients in the study population had received social assistance during at least one year of the study period. Based on Swedish data from 2012, 882,416 individuals received social assistance between 1991 and 2007. In a population of 9.1 million in 2007, that would correspond to approximately ten percent of the population. Hence, our study population received more social assistance. Longitudinal Swedish data suggest that the longer an individual receives social assistance, the greater the risk that they will continue to do so^22^. In 2016, 389 cases (3·6%) in our population received social assistance, compared to 2·7% of the total population^23^. This raises concerns regarding the patient population that are treated for CTS surgically, where a large proportion of the current patients received continued social assistance, indicating that this is an exposed and possibly vulnerable population.

Socioeconomic factors affected the total QuickDASH scores both before and after surgery. However, again, the change in QuickDASH remained constant when comparing groups, suggesting that the effect of the surgery was the same, but that more deprived patients perceive their disability as worse than patients who are not as deprived. Similar results were found when investigating primary shoulder arthroplasty^24^. In total knee arthroplasty, patients with lower socioeconomic status have more pain and lower function at the time of surgery than patients with a higher socioeconomic status^25^. One study comprising 121,983 patients in England found low socioeconomic status to be associated with worse outcomes after total hip or knee replacement^26^, and in a large Danish study, higher education and higher earnings were associated with lower mortality following hip fracture^27^. There is limited evidence of a positive association between psychosocial factors and CTS^28^. It is also possible that more deprived patients have more comorbidities, and that patients with lower socioeconomic status have more concomitant upper extremity conditions that may affect the result in QuickDASH, since QuickDASH is not disease specific. Future research could include, for example, studies on electrophysiology, to investigate whether socioeconomically deprived patients present at a later stage of the disease. To summarize, in accordance with previous studies on other musculoskeletal conditions, this study shows that socioeconomic factors affect how patients perceive their symptoms and disability in CTS.

### Limitations

The approximately one third response rate in HAKIR during the study period may have impacted on the representativeness of the data, thereby affecting interpretation. The response rate was at the same level as previously reported^10,29,30^, with no clear explanation for attrition. Response rates have improved over time since HAKIR started in 2010, but further improvement is needed. While we found no differences in response rates between men and women, they were better among older participants.

We had no data on cohabiting, which is common in Sweden. For reference, in 2013 14% of the Swedish population were living with a partner without being married or having a contract (data from Statistics Sweden). These patients were classified as single in our study, which could have led to an underestimation of the health benefits of being married or in a stable relationship.

Measuring earned income meant that measuring economic circumstances focused on labour market productivity. Our measure takes no account of other aspects of economic standing, such as wealth or capital income. We also had no data on childhood socioeconomic circumstances or neighbourhood socioeconomic conditions that might also influence an individual’s health^4^. We only evaluated whether the patient had a manual occupation or not, and a further evaluation of occupational complexity was outside the scope of this study.

Mental health, a variety of concomitant upper extremity conditions and patient comorbidity might affect the surgical results. However, we did not have access to such data, including elbow and shoulder diseases and posttraumatic conditions, that may affect QuickDASH results. This will be an interesting field for future research.

## Conclusion

Many patients who are treated for CTS with OCTR are socioeconomically deprived. We conclude that being a widow, having a lower education level, low earnings and immigrant status, being on sick leave and social assistance are associated with greater disability both before and after OCTR for CTS, but that these factors do not affect the relative improvement, i.e. change, in QuickDASH. Further research is needed to discover the specific factors that may improve treatment outcome, and whether concomitant conditions are more prevalent in the deprived patient.

## Supplementary Information

Supplementary Information.

Supplementary Tables.

## Data Availability

Public access do data is restricted by the Swedish Authorities (Public Access to Information and Secrecy Act; http://government.se/information-material/2009/09/public-access-toinformation-andsecrecy-act/), but data can be available for researchers after a special review that includes approval of the research project by both an Ethics Committee and the authorities’ data safety committees.
