# Supplementary material for: Socioeconomic factors predicting outcome in surgically treated carpal tunnel syndrome: a national registry-based study
Source: Sci Rep. 2021 Jan 28;11:2581. doi: 10.1038/s41598-021-82012-x (PMC7844239; doi:10.1038/s41598-021-82012-x)
Supplement: Supplementary file 1 — Supplementary Information. [file 41598_2021_82012_MOESM1_ESM.pdf]

Socioeconomic factors predicting outcome in surgically treated carpal tunnel syndrome – a national registry-based study

Malin Zimmerman[a,b*], Evelina Hall[a], Katarina Steen Carlsson[c], Erika Nyman[d,e] , Lars B. Dahlin[a,b]

a) Department of Translational Medicine – Hand Surgery, Lund University, Skåne University Hospital, Jan Waldenströms gata 5, S-205 02 Malmö, Sweden
b) Department of Hand Surgery, Skåne University Hospital, Jan Waldenströms gata 5, S-205 02 Malmö, Sweden
c) Department of Clinical Sciences, Malmö, Lund University, Lund
d) Department of Biomedical and Clinical Sciences, Linköping University, Linköping, Sweden.
e) Department of Hand Surgery, Plastic Surgery and Burns, Linköping University Hospital, Linköping, Sweden.
f) Department of Hand Surgery, Plastic Surgery and Burns, Linköping University, Linköping, Sweden

***Corresponding author:** Malin Zimmerman, malin.zimmerman@med.lu.se; +46708495632

Supplemental Table S1. Cases with carpal tunnel syndrome (CTS) treated with open carpal tunnel release (OCTR) divided by marital status.

	Unmarried (n=2588)	Married (n=5453)	Divorced (n=1840)	Widowed (n=825)	P-value
Sex, female	1727 (67)	3400 (62)[b]	1318 (72)[d]	684 (83)[f]	**<0·0001**
Age, years	44 [34-53]	57 [47-68][b]	58 [50-67][c]	81 [73-85][f]	**<0·0001**
Bilateral cases	438 (17)	876 (16)[ns]	580 (16)[ns]	122 (15)[e]	**0·025**
Earnings, 1000 SEK, mean/year	223 [104-290]	217 [123-292][ns]	185 [82-263][d]	107 [5-195][f]	**<0·0001**
Days in unemployment	8 [0-31]	0 [0-14][b]	0 [0-3][d]	0 [0-0][f]	**<0·0001**
Born outside Sweden	188 (7)	1009 (19)[b]	464 (25)[d]	101 (12)[e]	**<0·0001**
Paid sick leave, days/employed year	10 [3-32]	8 [1-32][b]	17 [3-56][d]	0 [0-17][f]	**<0·0001**
Ever received social assistance	985 (38)	1521 (28)[b]	875 (48)[d]	97 (12)[f]	**<0·0001**
Preoperative QuickDASH	45 [32-64]	45 [30-61][ns]	55 [40-65][c]	57 [45-73][ns]	**<0·0001**
Postoperative QuickDASH at 3 months	18 [11-36]	18 [9-34][ns]	27 [11-44][d]	30 [13-47][ns]	**<0·0001**
Postoperative QuickDASH at 12 months	11 [5-33]	11 [2-32][ns]	20 [5-39][ns]	30 [11-51][e]	**<0·0001**
Change in QuickDASH score 0-12 months	25 [11-40]	25 [11-41]	25 [11-41]	21 [9-39]	0·69

Data are median [IQR] or n (%). Data missing in 40 cases. Cohabitant status was not included in our data and these individuals are hence included in the not married group.

Bilateral surgery shown as number of patients. Kruskal Wallis test with subsequent Bonferroni corrections were used to calculate statistical significance.

Supplementary Table S2. Cases with carpal tunnel syndrome (CTS) treated with open carpal tunnel release (OCTR) divided by level of education.

	Primary (n=2336)	Upper secondary (n=3441)	Tertiary (n=4783)	P-value
Sex, female	1390 (60)	2233 (65)[b]	3415 (71)[d]	**<0·0001**
Age, years	64 [51-77]	56 [48-67][b]	51 [38-63][d]	**<0·0001**
Bilateral surgery	343 (15)	572 (17)[a]	782 (16)[ns]	**0·004**
Earnings, 1000 SEK, mean/year	130 [24-230]	204 [121-265][b]	233 [138-312][d]	**<0·0001**
Days in unemployment/year	0 [0-11]	1 [0-21][b]	0 [0-18][ns]	**<0·0001**
Born outside Sweden	522 (22)	435 (13)[b]	745 (16)[d]	**<0·0001**
Paid sick leave, days/employed year	8 [0-42]	12 [3-42][b]	7 [1-27][d]	**<0·0001**
Ever received social assistance	816 (35)	1248 (36)[ns]	1379 (29)[d]	**<0·0001**
Preoperative QuickDASH	57 [41-70]	52 [36-68][a]	48 [32-64][d]	**<0·0001**
Postoperative QuickDASH at 3 months	30 [11-52]	23 [11-45][ns]	20 [9-36][d]	**<0·0001**
Postoperative QuickDASH at 12 months	25 [5-52][a]	18 [5-43]	14 [5-32][c]	**<0·0001**
Change in QuickDASH score 0-12 months	25 [7-41]	27 [11-43]	25 [11-39]	0·39

Data are median [IQR] or n (%). [a]p<0·05 when compared to group 1, [b]p<0·0001 when compared to group 1, [c]p<0·05 when compared to group 2, [d]p<0·0001 when compared to group 2, ns= non-significant.

Bilateral surgery shown as number of patients. Kruskal Wallis test with subsequent Bonferroni corrections were used to calculate statistical significance.

Education level was divided into three groups. These groups correspond to The International Standard Classification of Education (ISCED) [16] as follows: primary: ISCED 0, 1 and 2 (≤9 years of education); upper secondary: ISCED 3 (9-12 years of education) and tertiary: ISCED 4, 5 and 6 (>12 years of education).

Supplementary Table S3. Cases with carpal tunnel syndrome (CTS) treated with open carpal tunnel release (OCTR) based on mean annual earnings.

	**≤98,100 SEK (n=2688)**	**98,101-202,600 SEK (n=2685)**	**202,601-281,000 SEK (n=2687)**	**>281,000 SEK (n=2686)**	**P-value**
Sex, female	2050 (76)	2035 (76)[ns]	1914 (71)[d]	1151 (43)[f]	**<0·0001**
Age, years	58 [42-76]	58 [48-72][a]	54 [45-64][d]	53 [42-63][e]	**<0·0001**
Bilateral cases	379 (14)	418 (16)[a]	473 (18)[c]	448 (17)[ns]	**<0·0001**
Highest education level	925 (34)	1011 (38)[a]	1233 (46)[d]	1614 (60)[f]	**<0·0001**
Days in unemployment/year	0 [0-26]	5 [0-30][b]	0 [0-16][d]	0 [0-8][f]	**<0·0001**
Born outside Sweden	835 (31)	409 (15)[b]	310 (12)[d]	213 (8)[f]	**<0·0001**
Paid sick leave, days/year	38 [0-126]	19 [3-57][ns]	8 [2-23][d]	3 [1-12][f]	**<0·0001**
Ever received social assistance	1188 (44)	979 (36)[b]	795 (30)[d]	522 (19)[f]	**<0·0001**
Preoperative QuickDASH	61 [48-75]	52 [36-68][b]	50 [34-66][ns]	43 [27-57][f]	**<0·0001**
Postoperative QuickDASH at 3 months	36 [16-59]	25 [11-43][b]	20 [9-36][c]	16 [8-30][d]	**<0·0001**
Postoperative QuickDASH at 12 months	34 [13-59]	18 [5-43][b]	14 [3-32][c]	9 [2-23][d]	**<0·0001**
Change in QuickDASH score 0-12 months	22 [6-41]	25 [11-43]	27 [14-41]	25 [11-36]	0·12

Data are median [IQR] or n (%). [a]p<0·05 when compared to group 1, [b]p<0·0001 when compared to group 1, [c]p<0·05 when compared to group 2, [d]p<0·0001 when compared to group 2, ns= non-significant

Data missing in 10 cases. Bilateral surgery shown as number of patients. Kruskal Wallis test with subsequent Bonferroni corrections were used to calculate statistical significance.

Earnings were adjusted by the consumer price index as of December 2016 (available from Statistics Sweden, www·scb·se). Data regarding earnings was available from 1990-2016. A binned variable was created, based on mean earnings per year above 30 and under 65 years of age.

Supplementary Table S4. Cases with carpal tunnel syndrome (CTS) treated with open carpal tunnel release (OCTR) based on migrant status.

	**Born in Sweden (n=8979)**	**Born outside of Sweden (n=1767)**	**P-value**
Sex, female	5854 (65)	1296 (73)	**<0·0001**
Age, years	56 [45-69]	54 [44-62]	**<0·0001**
Bilateral cases	1472 (16)	245 (14)	**<0·0001**
Highest education level	4038 (45)	745 (42)	**0·03**
Days in unemployment/year	0 [0-16]	4 [0-31]	**<0·0001**
Ever received social assistance	2461 (27)	1023 (58)	**<0·0001**
Paid sick leave, days/ employed year	8 [1-32]	17 [2-64]	**<0·0001**
Mean earnings/year, 1000 SEK	217 [123-291]	114 [3-225]	**<0·0001**
Preoperative QuickDASH	48 [32-64]	57 [39-70]	**<0·0001**
Postoperative QuickDASH at 3 months	20 [9-36]	27 [11-50]	**<0·0001**
Postoperative QuickDASH at 12 months	14 [5-32]	25 [7-50]	**<0·0001**
Change in QuickDASH score 0-12 months	25 [11-41]	25 [7-36]	0·36

Bilateral surgery shown as number of patients. Data presented as number (%) or median [interquartile range, IQR]. Mann Whitney U-test was used to calculate statistical significance.

Supplementary Table S5. Cases with carpal tunnel syndrome (CTS) treated with open carpal tunnel release (OCTR) based on mean payed sick leave days during years of employment.

	**0 days (n=1338)**	**1-9 days (n=2831)**	**10-35 days (n=2007)**	**>36 days (n=2059)**	**P-value**
Sex, female	685 (51)	1723 (61)[b]	1318 (66)[c]	1515 (74)[f]	**<0·0001**
Age, years	75 [66-82]	55 [47-64][b]	55 [47-63][ns]	57 [49-66][f]	**<0·0001**
Bilateral cases	203 (15)	453 (16)	333 (17)	331 (16)	**0·41**
Highest education level	541 (40)	1306 (46)[b]	851 (42)[c]	714 (35)[f]	**<0·0001**
Days in unemployment/ year	0 [0-0]	0 [0-15][b]	3 [0-23][d]	9 [0-32][f]	**<0·0001**
Born outside Sweden	131 (10)	249 (9)[ns]	225 (11)[c]	364 (18)[f]	**<0·0001**
Ever received social assistance	152 (11)	629 (22)[b]	694 (35)[d]	988 (48)[f]	**<0·0001**
Mean earnings/year, 1000 SEK	184 [81-307]	262 [211-326][b]	236 [182-290][d]	145 [80-209][f]	**<0·0001**
Preoperative QuickDASH	45 [64-27]	41 [27-59][a]	52 [39-66][d]	61 [48-75][f]	**<0·0001**
Postoperative QuickDASH at 3 months	23 [9-41]	16 [7-30][b]	24 [13-43][d]	33 [16-52][f]	**<0·0001**
Postoperative QuickDASH at 12 months	27 [7-48]	9 [2-23][b]	18 [5-36][d]	30 [10-52][f]	**<0·0001**
Change in QuickDASH score 0-12 months	19 [7-32]	25 [13-39][a]	30 [17-45][ns]	23 [9-43] [ns]	**<0·0001**

Data missing in 2511 cases. Sick leave calculated as net days exceeding 14 days during employed years. Bilateral surgery shown as number of patients. Data presented as number (%) or median [interquartile range, IQR]. Kruskal Wallis test with subsequent Bonferroni corrections were used to calculate statistical significance.

[a]p<0·05 between group 1 and 2, [b]p<0·0001 between group 1 and 2, [c]p<0·05 between group 2 and 3, [d]p<0·0001 between group 2 and 3, [e]p<0·05 between group 3 and 4, [f]p<0·0001 between group 3 and 4· ns=non-significant.

Supplementary Table S6. Outcome after OCTR and patient characteristics. Groups based on whether the patient ever received social assistance.

	**Never received (n=7262)**	**Received once (n=846)**	**Received multiple times (n=2638)**	**P-value**
Sex, female	4652 (64)	577 (68)[b]	1921 (73)[c]	**<0·0001**
Age, years	59 [48-72]	53 [41-62][b]	49 [40-57][d]	**<0·0001**
Born outside Sweden	744 (10)	137 (16)[b]	886 (34)[d]	**<0·0001**
Never employed	382 (5)	24 (3)[b]	278 (11)[d]	**<0·0001**
Highest education level	3404 (47)	373 (44)[ns]	1006 (38)[c]	**<0·0001**
Mean earnings/year, 1000 SEK	224 [125-299]	218 [135-278][ns]	143 [31-228][d]	**<0·0001**
Sick days/employed year	5 [1-24]	14 [3-48][b]	26 [8-80][d]	**<0·0001**
Days as unemployed/year	0 [0-8]	5 [0-21][b]	24 [2-47][d]	**<0·0001**
Preoperative QuickDASH	48 [32-64]	52 [36-68][a]	59 [43-73][c]	**<0·0001**
Postoperative QuickDASH at 3 months	20 [9-39]	20 [11-41][ns]	32 [16-55][d]	**<0·0001**
Postoperative QuickDASH at 12 months	14 [5-34]	16 [2-41][ns]	32 [11-57][d]	**<0·0001**
Change in QuickDASH score 0-12 months	25 [11-41]	23 [11-39]	27 [9-43]	0·82

Data presented as number (%) or median [interquartile range, IQR]· Bilateral surgery shown as number of patients. ns = non-significant. Kruskal Wallis test with subsequent Bonferroni corrections were used to calculate statistical significance.

[a]p<0·05 between group 1 and 2, [b]p<0·0001 between group 1 and 2, [c]p<0·05 between group 2 and 3, [d]p<0·0001 between group 2 and 3.

Supplementary Table S7. Multivariate linear regression analysis of factors of various socioeconomic status and the effects of the factors on QuickDASH score 12 months postoperative.

	**Model 1 Unadjusted B-coefficient (95% CI)**	**P-value**	**Model 2 Adjusted B-coefficient (95% CI) (adjusted for age, sex and diabetes)**	**P-value**	**Model 3a Adjusted B-coefficient (95% CI) All variables included**	**P-value**
Age at surgery	0·16 (0·094-0·22)	**<0·0001**	0·17 (5·1-14·0)	**<0·0001**	-0·024 (-0·19-0·14)	0·78
Sex	4·6 (2·4-6·8)	**<0·0001**	5·7 (3·5-7·9)	**<0·0001**	0·97 (-2·4-4·4)	0·58
Diabetes at surgery	3·3 (0·41-6·2)	**0·025**	3·1 (0·19-6·0)	**0·037**	2·1 (-1·9-6·1)	0·29
Marital status						
Not married (reference)						
Married	2·4 (-0·31-5·0)	0·083	1·08 (-1·8-3·9)	0·46	1·0 (-2·8-4·8)	0·60
Divorced	5·3 (1·9-8·7)	**0·003**	3·7 (0·088-7·3)	**0·045**	-1·3 (-6·0-3·4)	0·59
Widowed	11·8 (7·9-15·7)	**<0·0001**	7·3 (2·7-12·0)	**0·002**	8·4 (1·3-15·6)	**0·021**

Level of education						
Low (reference)						
Middle	-4·8 (-7·6- -2·1)	**0·001**	-4·5 (-7·2- -1·7)	**0·002**	-3·8 (-7·6-0·084)	0·055
High	-10·3 (-12·8- -7·7)	**<0·0001**	-9·6 (-12·3- -6·9)	**<0·0001**	-6·5 (-10·7- -2·3)	**0·003**
Earnings (mean/year)						
≤98,100 (reference)						
98,101-202,600	-11·3 (-14·1- -8·5)	**<0·0001**	-11·2 (-13·9- -8·4)	**<0·0001**	-8·0 (-13·0- -2·9)	**0·002**
202,601-281,000	-17·1 (-19·8- -14·3)	**<0·0001**	-16·4 (-19·2- -13·5)	**<0·0001**	-9·4 (-15·0- -3·8)	**0·001**
>281,000	-21·1 (-23·9- -18·4)	**<0·0001**	-19·8 (-22·7- -16·9)	**<0·0001**	-11·6 (-17·9- -5·3)	**<0·0001**
Migrant status						

Born in Sweden (reference)						
Born outside of Sweden	13·0 (10·1-15·9)	**<0·0001**	13·0 (10·1-15·9)	**<0·0001**	5·7 (1·3-10·0)	**0·01**
Occupation						
Non-manual (reference)						
Manual	4·0 (0·58-7·3)	**0·022**	3·6 (0·19-7·0)	**0·038**	-1·2 (-4·9-2·5)	0·52
Sick leave						
0 days (reference)						
1-9 days	-12·5 (-15·0- -10·0)	**<0·0001**	-11·6 (-14·0- -9·1)	**<0·0001**	-6·9 (-12·1- -1·6)	0·011
10-35 days	-4·5 (-7·3- -1·7)	**0·002**	-4·3 (-7·1- -1·5)	**0·002**	1·0 (-4·5-6·5)	0·72
>36 days	5·8 (2·9-8·7)	**<0·0001**	5·7 (2·8-8·6)	**<0·0001**	5·3 (-0·38-11·0)	0·067
Unemployment						

Mean days/year	0·048 (-0·007-0·10)	0·089	0·12 (0·063-0·18)	**<0·0001**	-0·018 (-0·097-0·060)	0·66
Social assistance						
Never received (reference)						
Received once	2·4 (-1·6- 6·4)	0·25	4·3 (0·3-8·2)	**0·034**	7·1 (1·6-12·7)	0·012
Received more than once	13·0 (10·4-15·6)	**<0·0001**	15·2 (12·6-17·9)	**<0·0001**	9·7 (5·8-13·5)	<0·0001

B-coefficients are unstandardized.

Sex – men is the reference category.

Supplementary Table S8. Reduced model 3b. Variables with a p<0·3 in the original model are included.

	**Unadjusted B-coefficient (95% CI)**	**P-value**
Diabetes at surgery	0·97 (-1·7-3·6)	0·48
Marital status		
Not married/married/divorced (reference)		
Widowed	5·9 (2·6-9·1)	**<0·0001**
Level of education		
Low (reference)		
Middle	-2·0 (-4·6-0·7)	0·14
High	-5·0 (-7·5- -2·5)	**<0·0001**
Earnings (mean/year)		
≤98,100 (reference)		
98,101-202,600 SEK	-7·9 (-10·7- -5·1)	**<0·0001**
202,601-281,000 SEK	-10·2 (-13·2- -7·3)	**<0·0001**
>281,000 SEK	-12·9 (-15·0- -8·9)	**<0·0001**
Migrant status		
Born in Sweden (reference)		
Born outside of Sweden	7·3 (4·4-10·1)	**<0·0001**
Sick leave		
0 days/10-35 days (reference)		
1-9 days	-6·1 (-8·4- -3·8)	**<0·0001**
>36 days	5·3 (2·6-7·9)	**<0·0001**
Social assistance		

Never received (reference)		
Received once	1·8 (-2·0-5·5)	0·36
Received more than once	8·2 (5·6-10·8)	**<0·0001**

Excluded variables: age at surgery, sex, manual/non-manual occupation and unemployment.
