# Supplementary material for: Socioeconomic factors predicting outcome in surgically treated carpal tunnel syndrome: a national registry-based study
Source: Sci Rep. 2021 Jan 28;11:2581. doi: 10.1038/s41598-021-82012-x (PMC7844239; doi:10.1038/s41598-021-82012-x)
Supplement: Supplementary file 2 — Supplementary Tables. [file 41598_2021_82012_MOESM2_ESM.pdf]

	**Model 1 Unadjusted B-coefficient (95% CI)**	**P-value**	**Model 2 Adjusted B-coefficient (95% CI) (adjusted for age, sex and diabetes)**	**P-value**	**Model 3a Adjusted B-coefficient (95% CI) All variables included**	**P-value**
Age at surgery	0.129 (0.077-0.18)	**<0.0001**	0.14 (0.09-0.19)	**<0.0001**	0.10 (-0.05-0.25)	0.2
Sex	5.37 (3.6-7.1)	**<0.0001**	6.4 (4.6-8.1)	**<0.0001**	2.0 (-0.76-4.8)	0.15
Diabetes at surgery	4.8 (2.5-7.1)	**<0.0001**	4.8 (2.5-7.1)	**<0.0001**	-0.11 (-3.6-3.4)	0.95
Marital status						
Not married (reference)						
Married	-0.5 (-2.6-1.6)	0.6	-1.6 (-3.8-0.60)	0.15	-0.87 (-3.9-2.2)	0.58
Divorced	4.5 (1.8-7.1)	**0.001**	2.8 (-0.004-5.5)	0.05	-1.8 (-5.7-2.0)	0.8
Widowed	8.8 (5.6-12.1)	**<0.0001**	4.1 (0.31-7.89)	**0.034**	1.7 (-6.0-9.3)	0.67

Level of education						
Low (reference)						
Middle	-3.2 (-5.4—0.97)	**0.005**	-2.86 (-5.1--0.65)	**0.011**	-0.54 (-4.1 – 3.0)	0.77
High	-8.0 (-10.0—5.9)	**<0.0001**	-7.3 (-9.5--5.2)	**<0.0001**	-3.2 (-6.7-0.28)	0.07
Earnings (mean/year)						
≤98,100 (reference)						
98,101-202,600	-4.7 (-5.8—3.6)	**<0.0001**	-4.7 (-5.8--3.5)	**<0.0001**	-3.2 (-5.9—0.5)	**0.02**
202,601-281,000	-4.1 (-4.9—3.4)	**<0.0001**	-4.2 (-5.0--3.4)	**<0.0001**	-1.8 (-3.6-0.05)	0.057
>281,000	-3.9 (-4.5- -3.4)	**<0.0001**	-3.7 (-4.3--3.1)	**<0.0001**	-1.1 (-2.5-0.28)	0.12
Migrant status						
Born in Sweden (reference)						
Born outside of Sweden	12.0 (9.6-14.3)	**<0.0001**	11.9 (9.6-14.3)	**<0.0001**	7.5 (3.4-11.6	**<0.0001**
Occupation						

Non-manual (reference)						
Manual	1.3 (-0.96-3.6)	0.26	1.4 (-0.84-3.7)	0.22	-1.6 (-4.1-0.89)	0.2
Sick leave						
0 days (reference)						
1-9 days	-9.6 (-11.6--7.6)	**<0.0001**	-8.7 (-10.7- -7.0)	**<0.0001**	-2.7 (-7.0-1.7)	0.23
10-35 days	-2.0 (-4.3-0.21)	0.077	-1.8 (-4.1-0.40)	0.11	3.4 (-1.2 – 8.0)	0.14
>36 days	6.0 (3.6-8.3)	**<0.0001**	5.5 (3.2-7.8)	**<0.0001**	9.7 (4.6-14.8)	**<0.0001**
Unemployment						
Mean days/year	0.08 (0.04-0.12)	**<0.0001**	0.13 (0.084-0.17)	**<0.0001**	0.08 (0.013-0.15)	**0.02**
Social assistance						
Never received (reference)						
Received once	2.5 (-0.5-5.6)	0.11	3.8 (0.80-6.8)	**0.013**	2.9 (-1.2-7.0)	0.17

Received more than once	10.9 (8.88-13.0)	**<0.0001**	12.5 (10.4-14.6)	**<0.0001**	5.5 (2.2-8.8)	**0.001**

Dependent variable: QuickDASH at 3 months postop
